# Development and retention of the dental workforce: findings from a regional workforce survey and symposium in England

**DOI:** 10.1186/s12913-020-4980-6

**Published:** 2020-03-26

**Authors:** Richard D. Holmes, Bryan Burford, Gillian Vance

**Affiliations:** 1 School of Dental Sciences, Newcastle University, Framlington Place, Newcastle upon Tyne, NE2 4BW UK; 2 School of Medical Education, Faculty of Medical Sciences, Newcastle University, Cookson Building, Newcastle upon Tyne, NE2 4HH UK

**Keywords:** Dentistry, Workforce, Survey, Contract reform, Skill-mix, Quantitative, Qualitative

## Abstract

**Background:**

To help promote a flexible and sustainable workforce in dentistry, it is necessary to access accurate and timely data about the structure and nature of the evolving dental team. This paper considers the results and learning from a region-wide dental workforce survey conducted in one area of Health Education England and how the team has changed since the last survey a decade earlier.

**Methods:**

A mixed-methods approach comprised two phases. In Phase 1 a customised workforce questionnaire was sent to all dental practices registered with the Care Quality Commission in the North East of England and North Cumbria in March 2016. Findings then informed Phase 2, a regional symposium held in October 2016, where interactive workshops generated qualitative data that elaborated on factors influencing workforce development.

**Results:**

Of 431 primary dental care practices identified, 228 questionnaires were returned - a 53% response rate. The largest professional groups were dental nurses (*n* = 1269, 53% by headcount; 50% of fte) and dentists (34% by headcount; 42% by fte), though there had been increases in numbers of all staff groups over the decade, which was most marked for dental therapists (from 1 per 39 dentists to 1 per 8 dentists). The dental team predominantly fell into ‘younger’ age groups (< 46 years age), with evidence of a significant increase in the number of dentists reporting part-time working in a practice since the last survey. Around one third of dental practices reported employing dental nurses with additional skills (*n* = 74, 32.5%) or dental therapists (*n* = 73, 32%), and nearly half employed a dental hygienist (*n* = 104, 46%). However, there was considerable variability in whether these staff actually carried out the range of skills within their scope of practice. Factors shaping workforce development were identified as, the national context, loss of expertise, patients’ health needs and expectations, surgery premises and financial constraints.

**Conclusions:**

The composition and work patterns of the primary care dental workforce have changed markedly over the last decade, though utilisation of skill-mix continues to be constrained. Consideration of factors determining career progression of dentists and dental care professionals is needed to optimise a sustainable future workforce.

## Introduction

Dental services across the UK are operating in a changing clinical, social and political climate [1, 2]. The incidence of some dental diseases, such as dental caries and periodontal disease in adults, may be falling [3]. However, among the ageing population who are retaining their natural teeth for longer, increasing numbers of patients have multiple co-morbidities and complex oral health needs [4]. Further, there are recognised geographical inequities of oral health in the UK, and of patients’ access to dental care [2, 5, 6]. This national context supports a case for reform of the dental workforce, with greater use of an integrated multi-disciplinary team, shaped to address the relevant needs of the population [7].

The concept of ‘skill-mix’, or broadly, ‘people with the right skills doing the right jobs’, is long established. It is over 25 years since the Nuffield Foundation advocated use of dental auxiliaries to support and rationalise the work of dentists [8]. A systematic literature review then concluded that appropriately trained ‘professionals complementary to dentistry’ were as competent as dentists in screening, diagnosis and a range of procedures [9]. Analysis of patient records in practices in Wales identified that at least one third of routine dentistry in primary care could be provided by hygienists and therapists [10] (though at that time they had a more limited scope of practice which has since been revised and expanded by the UK’s General Dental Council (GDC)).

The term ‘dental care professional’ (DCP) encompasses dental nurses, dental hygienists and dental therapists, who are also regulated by the GDC alongside dentists. These professional groups, along with others, were first subject to compulsory registration in 2008, and in 2009 the GDC produced new guidance (updated in 2013) which included examples of ‘additional skills’ that might be developed by DCPs in order to enhance their Scope of Practice [11]. The term ‘Extended Duties Dental Nurses’ (EDDNs) acknowledges those dental nurses who have developed their skills, while ‘Direct Access’ arrangements now allow some DCPs to carry out certain interventions without the patient having to see a dentist first [12]. These developments in the regulation and expansion of the scope of practice of DCPs have significant implications for dental workforce planning.

The anticipated advantages of a flexible workforce include increased efficiency of delivery, ease of patient access, and cost-effectiveness of care. Further long-term benefits may be associated with increasing amounts of preventative dentistry being carried out in primary care [13, 14]. However, while there is growing examination of skill-mix in primary medical practice [15] and emerging evidence of benefit [16], there is little literature in dentistry around how the dental workforce is changing in contemporary models of care.

In considering skill-mix, there are two key challenges. The first relates to an adequate supply of suitably trained DCPs [17, 18], while the second relates to how they are deployed in the workplace. The literature suggests that optimal use of skill-mix may be constrained with negative effects on work experiences of DCPs [19]. Limiting factors have included poor awareness and acceptability of the role among dentists [18, 20]. Other issues cited are a need for training of dentists themselves, such that they are equipped to lead a multi-disciplinary, integrated team [21] and the perennial challenge of the terms of the existing National Health Service (NHS) general dental services (GDS) contract in England, which largely measures clinical productivity through ‘units of dental activity’ (UDAs) [22–24].

Thus, as healthcare systems continue to evolve [25], questions examining workforce planning in dentistry and the challenges of enabling new ways of working are particularly salient to practitioners, and those individuals involved in training and the organisation of services.

While workforce planning is typically based on a need for a stable workforce composition, the recent policy and regulatory changes mean that this cannot be assumed. Hence, an up-to-date understanding of the changing structure and nature of the dental workforce can provide more information than simply registrant numbers.

This paper reports findings from a regional dental workforce survey (DWS) and workshop event in 2016, commissioned by Health Education England working across the North East and North Cumbria (HEE-NENC). Our study gathered data across different sectors (primary and secondary care, community (salaried) services and prison settings, dentists and DCPs employed in higher education institutions [HEIs]), but in this paper our focus is on general dental practice in primary care where the vast majority of dental care is delivered and changing skill-mix is arguably most pertinent. Whilst the initial survey report prepared for HEE is available online [26], in this paper we additionally explore factors that influence development of the dental team, based upon perceptions of a range of stakeholders involved. Relatively little is known about the composition and integration of professional groups within the dental workforce at a regional level in England. Consequently, this paper informs the literature by describing the shape of the dental workforce in a large geographic region in the north of England in 2016.

### Study rationale and aims

The study set out to provide the HEE local office with baseline data to inform development of a regional dental workforce strategy. Such data had not been collected in this region since 2006. In this paper we address two main aims. Firstly, to describe the composition of the regional dental workforce and how teams based in primary care have changed over the last 10 years. Secondly, to examine the factors affecting workforce development from the perspectives of dental professionals in the region.

## Methods

The study was designed in two consecutive phases.

Firstly, a questionnaire was devised, piloted and distributed to all dental care settings region-wide to determine the structure and composition of the dental workforce. Secondly, qualitative data was collected during a large regional dental workforce symposium involving dentists and DCPs in order to elaborate upon findings from the survey.

### Phase 1: dental workforce survey (DWS)

#### Scope and sample

The study was designed to include all practices, hospitals and services where clinical dental care is delivered to patients – this included NHS, private and mixed NHS/private services. The geographical scope was limited to North East England and North Cumbria (the area of responsibility held by the local office of Health Education England).

Sites were identified from publicly available registration data held by the Care Quality Commission (CQC). All physical locations in which health or social care is delivered in England must be registered with the CQC, and so this provided the most comprehensive and up-to-date record of all settings, delivering NHS and private dental care. The sample was identified by limiting the CQC database to locations delivering dental services in the ‘services’ field, and geographically using ‘region’ and ‘postcode’ fields.

As outlined above, this paper focuses on data from primary care, where 431 practices were identified, but for completeness, data were also provided by 4 hospital Trusts, a Lead Employer Trust, a service provider for a group of 7 prisons and 6 HEIs across the North East and North Cumbria.

#### Data collection tool

A questionnaire was developed to collect basic demographics and the professional composition of the dental workforce. This requested data on current numbers of staff occupying different professional groups, their ages, qualifications and current use of enhanced skills by DCPs. In addition, it sought information on staff vacancies, the type of provider (single owner, independent partnership, corporate) and whether the practice provided NHS or private services only, or a mixture of both. Data were predominantly quantitative, but free text comments about perceived staff development needs and any other relevant workforce information were also invited. The final version of the questionnaire for primary dental care is provided in ‘Additional file 1’.

Numbers of staff were captured both as headcount – the number of people employed – and full time equivalent (fte) – the number of full time posts represented by those people. The fte number indicates workload, and capacity, while headcount represents the actual size of the workforce. Comparison of the two indicates the extent of less-than-full-time working, from which workforce preferences can be inferred.

The questionnaire was designed for completion in primary dental care, with other versions for different organisational settings adapted from this base. In order to establish face validity of the tool, it was reviewed by members of a project advisory group (PAG) and piloted prior to distribution.

#### Distribution

Dental practices were contacted by email, where possible, in advance of questionnaire distribution, in order to raise awareness of the study. In early March 2016, the survey was distributed by post. Envelopes were marked with the Newcastle University logo, the strapline ‘Dental Workforce Survey 2016’, and the questionnaire was printed on coloured paper to differentiate it from routine paperwork. All paper questionnaires were accompanied by a letter from the authors and a guidance document from the regional Postgraduate Dental Dean explaining the purpose of the requested information. A return stamped-addressed envelope was included in the package along with a web-link to an online version of the questionnaire so that practices could complete the questionnaire online if they preferred. A unique identifier was included on each questionnaire, and this was required in the online version to avoid duplicate returns.

Email reminders were cascaded to non-responders, and a second paper copy was mailed 5 weeks after the initial distribution. After a further month, a final follow-up phone call from one of the project team invited non-responders to submit the survey by post, online, or verbally - directly with a researcher.

### Phase 2: workshops

**Fig. 1:**
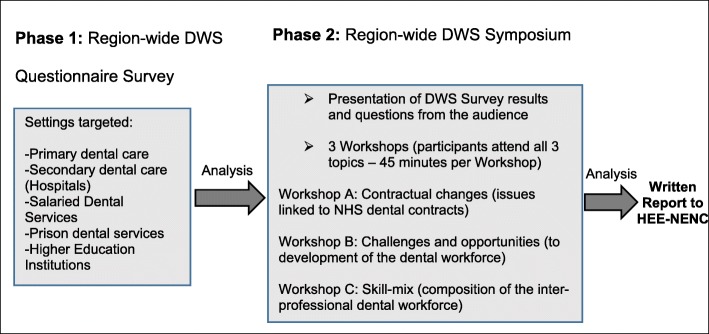
Overview of the study design. The study was conducted in two phases: a region-wide questionnaire survey, and a one day symposium, which sought to elaborate findings from the survey. (DWS - Dental Workforce Survey, HEE-NENC - Health Education England working across the North East and North Cumbria)

Data in Phase 2 were collected through small group workshops conducted as part of a regional symposium on dental workforce strategy. Initial presentation of the questionnaire findings was followed by a series of three workshops based around key issues informed by the quantitative data. All participants discussed the three topics in a different order. The three workshop topics are listed in Fig. 1. Each used a separate topic guide to structure the discussions.

Workshops lasted 45 min and each was facilitated by a member of the project team alongside a member of the HEE local office postgraduate dental training team. The pairings were designed to ensure a combination of research and domain expertise.

Each workshop was held in a separate conference room with up to 12 delegates representing diverse dental professional groups and incorporating various lengths of professional experience and NHS-private provider mix. Participants were asked to nominate a scribe to record key points on flip charts and they were encouraged to provide anonymous comments on self-adhesive notes. At the close of the symposium, the flipcharts and attached notes from delegates were collated alongside the authors’ personal notes. All materials were coded and summarised following principles of thematic analysis – we did not aim to reduce data simply to content analysis, but by comparison between groups and reference to our own field notes, to develop a richer picture of participant views.

#### Ethical review

NHS research ethics approval was not required as the work was commissioned as a service evaluation and involved only data collection from staff participants. Preliminary review by Newcastle University Faculty of Medical Sciences Research Ethics Committee confirmed that full review was not required for this study. Neither dental practices nor individual participants are identified in this paper.

#### Project advisory group

At the outset of the study, the authors formed a Project Advisory Group (PAG) to provide feedback and guidance from the perspective of potential respondents. Interested dental professionals were invited to participate with the PAG via email, through HEE and by word of mouth. The final group of 8 clinicians included NHS and private dentists, with representation from primary and secondary care (hospital services) and prison dental services. PAG involvement was mainly via face-to-face meetings with the authors, however email correspondence was also welcomed from those unable to attend. The PAG advised upon aspects such as questionnaire format, the intelligibility of terminology used within questions, methods for information cascade, including optimum timing within the calendar year for questionnaire dissemination and use of a monetary incentive (prize draw) to support participation.

#### Data analysis

Data entry from paper questionnaires was undertaken by the authors, while online responses were downloaded and imported directly into a spreadsheet. Frequencies and descriptive statistics were calculated for numerical data, including Chi-square statistics to test for differences in categorical variables. Content analysis of the open-ended textual questions was used to identify preferences for the local programme of continuing dental education.

The quantitative findings from the questionnaire provided the basis for phase 2 of the project.

## Results

### Phase 1 – regional dental workforce questionnaire

#### Response rates

Following the planned sequence of reminders, the questionnaires achieved an overall response rate of 53% from primary dental care practices (228 of 431 practices across the region). Three quarters of these questionnaires were returned by post (*n* = 172) and the remainder (*n* = 56) were submitted online. The response rate from different types of providers was broadly similar (corporate bodies 46% (33/72), independent partnerships 56% (35/63) and single owner practices 54% (160/296)), suggesting that there was no systematic response bias between these groups. Additionally, more than 40% of practices in each of seven postcode areas in the region responded, indicating representation of the workforce in differing socio-economic neighbourhoods.

#### Size of workforce

As noted, there are two figures to consider in the size of the workforce, headcount and full time equivalent (fte). Headcount is the primary figure to attend to for training, as this is the number of people in the workforce. Fte is, however, an indicator of workload and the capacity of the system.

To allow comparison, the data for all professional groups and settings are listed in Table 1. For all staff groups, primary care is the largest employing sector, comprising 79% of the entire workforce by headcount, and 77% by fte. For some DCPs, the proportion was up to 100%. For consistency, remaining data refer only to the primary care workforce.

**Table 1:** Size of regional dental workforce

Professional Group	Primary Care		Secondary Care		Salaried		Prison		Total	
	h/c (% of total)	fte (% of total)	h/c (%)	fte (%)	h/c (%)	fte (%)	h/c (%)	fte (%)	h/c	fte
Dentists	804 (70.7)	561 (74.8)	219 (19.2)	117 (15.6)	107 (9.4)	70 (9.3)	8 (0.7)	1.5 (0.2)	1138	749.5
Dental nurses	1090 (79.9)	557 (72.7)	141 (10.3)	109 (14.2)	128 (9.4)	95.5 (12.5)	6 (0.4)	4.4 (0.6)	1365	765.9
Dental nurses in training	179 (98.9)	113 (98.5)	0	0	1 (0.6)	1 (0.9)	1 (0.6)	0.7 (0.6)	181	114.7
Dental therapists	99 (86.8)	39 (88.8)	0	0	8 (7.0)	3.5 (8.0)	7 (6.1)	1.4 (3.2)	114	43.9
Dental hygienists	158 (89.3)	40 (81.6)	13^a^ (7.3)	6 (12.2)	6 (3.4)	3 (6.1)	0	0	177	49
Dental technicians	18 (35.3)	16 (37.2)	31 (60.8)	25 (58.1)	2 (3.9)	2 (4.7)	0	0	51	43
Clinical dental technicians	10 (100)	3 (100)	0	0	0	0	0	0	10	3
Orthodontic therapists	22 (100)	10 (100)	0	0	0	0	0	0	22	10

Table 1 Survey figures listing headcount (h/c), full-time equivalent (fte) commitment and relevant percentage of total workforce for different dental professional groups. (^a^ = includes joint therapist/hygienist roles)

Dental nurses (both registered and pre-registration) comprised the single largest professional group (*n* = 1269, 53% of the primary care workforce by headcount, *n* = 670, 50% of fte). Dentists comprised 34% of the primary care workforce by headcount, and 42% by fte. Other DCPs comprised less than 10% each of the workforce.

There were relatively few dental practices that provided solely NHS care (18 of 228, 7.9%) and many of these (10 of 18) had a single dentist (range 1–6 dentists). The majority of practices offered both NHS and private services (*n* = 179, 79%) and comprised up to 15 dentists (modal number of dentists = 3). Dental practices owned by corporate bodies tended to be slightly larger than practices operated by partnerships (average 4.4 dentists compared to 4.2 dentists per practice, respectively). Conversely, single owner dentists reported an average of 2.8 dentists working within their practices. Three quarters of dentists reported delivering ≥80% commitment to the NHS.

#### Demographics

Dentists in the survey were predominantly male (58%, *n* = 455). Most dentists were under 46 years’ age (529, 68%), with only 11% (85 of 778) of dentists being over 55. Chi-square statistics indicated that the age distribution for male and female dentists differed significantly (chi-square (3, *n* = 764) = 24.531, *p* < 0.0001). Examination of the distributions suggests this was due to a greater proportion of male dentists in the older age groups (for those under 30 years the male:female ratio was 52:48, but for those over 55 years this was 79:21).

Almost two thirds of dentists (64%, *n* = 513) were reported to have obtained their primary dental qualification in the North East of England, with 21% qualifying elsewhere in the UK and 5% qualifying elsewhere in Europe.

In the DCP groups, 98% of both registered dental nurses (*n* = 1067) and dental nurses-in-training (*n* = 179) were female. Notably, 90% of all dental nurses were under 46 years of age. Almost nine in 10 dental nurses gained their primary dental qualification in the North East of England, with just 6% (*n* = 66) qualifying elsewhere in the UK, and less than 1% (*n* = 2) qualifying elsewhere in the European Economic Area.

The remaining DCP groups collectively comprised just 13% (*n* = 307) of the primary care workforce identified in the survey. They included dental hygienists (*n* = 158), dental therapists (*n* = 99), dental technicians (*n* = 18), orthodontic therapists (*n* = 22) and clinical dental technicians (*n* = 10). All orthodontic therapists, 98% of dental hygienists and 97% of therapists were female and the majority of DCP groups first qualified in North East England (including 93% of dental hygienists and 78% of dental therapists). With regard to the age profile of DCP groups, all orthodontic therapists, 96% of dental therapists, and 64% of dental hygienists were below the age of 46 years. For the relatively small number of dental technicians included in the survey (*n* = 25) and working in primary care, just over half were < 46 years of age, but only 2% were female.

#### Full-time and part-time working

Full-time working was defined as 37.5 h per week. Within individual practices, only 118 (14.7%) primary care dentists worked at least this amount, although the questionnaire was unable to track individuals who may have worked across multiple premises. Both male and female dentists tended to work less than this, with female dentists reporting a mean of 26 h per week (std dev, 9.6 h), and male dentists 28 h per week (std dev, 11.4 h). There was a small difference in the proportion of male (82%) and female (88%) dentists who worked part-time (chi-square (df = 1, *n* = 759) = 3.997, *p* = 0.0455). Whilst detailed working hours were not recorded for DCPs in order to reduce the time burden for questionnaire respondents, the difference between headcount and fte figures shown in Table 1 suggests large numbers of DCPs are working less-than-full time.

#### Skill-mix

Skill-mix was also examined with respect to the professional composition of practices and how skill sets of DCPs were being used. Around one third of dental practices (*n* = 74, 32.5%) reported employing dental nurses with additional formal training (‘Extended Duties Dental Nurses’ EDDNs), amounting to almost one fifth of dental nurses (*n* = 197, 18%). A similar proportion of practices employed at least one dental therapist (*n* = 73, 32%), and nearly half employed a dental hygienist (*n* = 104, 46%). Very few practices employed any of, clinical dental technician, dental technician or orthodontic therapist (3, 3, 4% of practices, respectively). Within the EDDN group, the most common additional qualification was in the area of dental radiography (15% of dental nurses), followed by dental sedation nursing and oral health education (both 12%).

Table 2 shows the extent to which specific additional activities are undertaken by DCPs in their respective dental practices. There was considerable variability in whether the DCP group actually carried out tasks that are within their scope of practice. For example, only 43% of practices employing EDDNs indicated that these staff ‘applied fluoride varnish to teeth’, while 72% of practices employing dental hygienists reported that that group ‘delivered oral health education’. None of the activities were performed by the relevant DCP group in all responding practices, suggesting that the full skill set of DCPs is being under-used in this region.

**Table 2:** Specific activities undertaken by DCP groups in employing practices

	Dental Therapist (*n* = 73)	Dental Hygienist (*n* = 104)	EDDN (*n* = 74)	Clinical Dental Technician (*n* = 8)	Dental Technician (*n* = 7)	Orthodontic Therapist (*n* = 10)
Prescribe radiographs	12 (16%)	10 (10%)	4 (5%)	0	0	0
Take radiographs	44 (60%)	22 (21%)	31 (42%)	0	0	5 (50%)
Apply fluoride varnish to teeth	61 (82%)	61 (59%)	32 (43%)	0	0	3 (30%)
Deliver Oral Health Education	60 (81%)	75 (72%)	35 (48%)	1 (12%)	0	5 (50%)
Take impressions	48 (65%)	24 (23%)	27 (36%)	3 (38%)	0	8 (80%)
Give smoking cessation advice	53 (72%)	61 (59%)	19 (26%)	0	0	2 (20%)
Measure and record plaque indices	59 (80%)	78 (75%)	5 (7%)	0	0	3 (30%)
Administer inhalation sedation	3 (4%)	1 (1%)	0	0	0	0
Rubber dam	9 (12%)	2 (2%)	3 (4%)	0	0	0
Cannulation	0	0	1 (1%)	0	0	0

Table 2 This shows the number of practices who report employing DCP groups, and the proportion of those practices who indicate that the group carries out the specific activity ‘at any time’. Note, some of the duties listed may be out with the relevant group’s GDC scope of practice. We suspect this may be user error (e.g. if the form was completed online or perhaps submitted by a non-clinical member of the dental practice team). However, all responses are included here for completeness

Survey data identified 99 dental therapists in primary care (headcount), amounting to one dental therapist per 8 dentists. However, 65 of 73 (89%) practices employing dental therapists estimated that much of their therapist’s time was spent undertaking work traditionally associated with the scope of practice of a dental hygienist.

#### Changes since 2006

The previous regional survey was undertaken in 2006 before much of the regulatory change described earlier. We therefore compared responses to establish how the workforce may have developed in that period. While practices cannot be identified, the sample size of the earlier survey was similar to ours (210 of 426 practices, a 49% response rate).

There have been increases in numbers of all staff groups in the 10 year period, with the most marked increases being in the number of dentists (with a 37% increase in headcount from 587 to 804), dental nurses (84% increase, from 591 to 1090) and dental therapists (725% increase, from 12 to 99). Whilst the ratio of nurses to dentists was more or less stable (1:5 to 1:4), there was a striking increase in the proportion of therapists (from 1 per 39 dentists to 1 per 8 dentists).

Another notable change was in the increase in part-time working, particularly among men. In 2006 34% of male dentists reported working less-than-full time in a practice, a figure that had increased to 82% in 2016 (chi-square (1, *n* = 777) = 184.37, *p* < 0.0001). Whilst this figure does not account for dentists working across two (or more) practices, or who may have an additional role out with the practice, notwithstanding, the finding suggests that current dentists may be choosing different patterns of working.

Low numbers of dentists in older age categories was evident at both time points, and chi-square statistics comparing age distributions across years were non-significant for both men and women. In 2006, 35% of men and 18% of women were over 45 years’ age, while in 2016, 38.6% of men and only 23% of women were over 46 years’ age.

#### Summary

The DWS findings identify a large, multi-professional dental workforce operating across NHS and private dental care settings in the North East of England and North Cumbria. The vast majority of the workforce operate in primary dental care, and here dental nurses comprise the largest professional group (by headcount). There is a sharp decline in older staff across all groups, with nine in 10 dental nurses being less than 46 years’ age suggesting that many exit the profession early. In primary care, there appears to have been a significant increase in part-time working amongst the vast majority of dentists.

Only around one fifth of dental nurses were reportedly trained in additional skills, despite regulatory changes which permit their development. Among all DCPs, the extent to which skill-mix has been adopted across the region within primary dental care appears limited and variable.

### Phase 2 – dental workforce symposium

The second phase of this study reports the findings from workshops held at a regional symposium which were used to elaborate on the findings of the regional survey and understand factors that influence workforce development.

#### Factors influencing workforce development

##### *National context*

The survey illustrated the scale of the primary care dental workforce in this region, compared to other settings. Reflecting this, a widely expressed view was that relatively little dentistry needs to be performed within a hospital environment and participants were cognisant of national policy promoting integrated healthcare systems. As a counter point though, several hospital consultants stressed that such developments were not without consequence to secondary care settings, where a shift of dental care away from hospitals ran the risk of impacting negatively upon funding streams and the availability of suitable patients for postgraduate dental teaching.

However, while the philosophy of care ‘close to home’ was generally accepted, participants identified a number of challenges that they felt hampered development of new models of delivery.

##### *Loss of expertise*

The first challenge related to the striking survey finding that the regional primary dental workforce is largely less than 46 years of age. These observations generated widespread concern about a loss of expertise due to a lack of retention of the older – and presumably more experienced – dental professionals. A lack of career development opportunities was thought to explain the small number of dental nurses in the more senior age groups. Dental nurses, in particular, can have limited career progression, which may negatively affect their morale and retention as they become more senior. Further, the finding that many younger GDPs and DCPs are adopting flexible or part-time working patterns was thought to reflect a shift in attitudes to a healthier work-life balance compared to older colleagues.

With a more limited pool of expertise, there may be implications for the range of treatments that are provided in primary care. This is in the context of participants noting that more patients are being referred on already to salaried dental services for treatment because of constraints linked to the NHS dental contract. Further, they also felt that younger dentists lack a financial incentive to ‘upskill’ under the current arrangements for remuneration particularly within the NHS, which could also compound the situation.

##### *Patient health needs and expectations*

The second challenge reflected patient factors. Participants generally felt that population changes and associated clinical needs - increasing patient co-morbidities with more complex medical and dental conditions – were a significant driver to develop the dental workforce, including appropriate use of skill-mix. However, while there was broad agreement with the principle of dentists continuing to manage more complex cases, and appropriately trained DCPs managing more routine patients alongside measures for disease prevention and oral health promotion interventions, they also raised two areas of caution. First, was the competence of newly-qualified dentists to manage patients with complex treatment needs, as some felt that dental students had received relatively limited experience in undergraduate programmes. Second, several participants felt that some patients may be reluctant to receive a treatment from a DCP, which had been traditionally performed by a dentist. Many felt that patients are unlikely to be aware of the scope of practice of dental therapists (which could include a common treatment such as the placement of permanent fillings in adult teeth), which may lead to uncertainty about qualification and competence. However, there was little evidence offered in workshops that these concerns had actually been realised or voiced by patients in practice. Indeed, in a small number of NHS dental practices where dental therapists had been employed for some time, integration and efficiency of the dental team was reported as having been particularly positive for all those involved, including patients and dentists.

#### Surgery accommodation

Another challenge was the practical constraints of limited surgery space and dental chairs needed for additional activity. Small one or two chair practices had much less flexibility than larger practices to accommodate EDDN’s and therapists wishing to work at the same time as general dental practitioners. Participants acknowledged that even non-clinical interventions (e.g. the provision of smoking cessation advice delivered by an appropriately-trained dental nurse) required a private consultation space and that many practices simply did not have suitable space available.

#### Financial issues

Finally, a common concern among participants centred on the business model underpinning primary care dentistry, and, in particular, the system of remuneration associated with the 2006 NHS dental contract in England. At the time of the symposium, details about the reformed contract were being tested and much of the finer contractual detail was still to be announced. However, it appeared to cause significant tension, and constrained many practitioners’ abilities to plan and fully embrace skill-mix.

Most NHS contract holders flagged a risk of employing dental therapists when their business relied on achieving UDA targets, and the cost-effectiveness of DCPs in this system was uncertain. Examples were offered whereby tensions had developed between associate dentists ‘competing’ for core clinical activities with dental therapists in the same practice in order to earn sufficient UDAs to meet their annual NHS contractual targets. Some delegates who were associate dentists were reluctant to delegate treatments to DCPs as they would take the ‘financial hit’.

More generally, participants also saw the 2006 contract as being a specific barrier to workforce development given its current focus on *treatment activity*. They thought that oral health promotion was an ideal platform on which to further integrate DCPs in the clinical management of patients, but the level of NHS remuneration attached to the provision of many oral and dental disease prevention interventions was an ongoing stumbling block to the use of skill-mix in many primary care businesses.

## Discussion

### Key findings

This study has highlighted a number of important issues affecting development of a regional dental workforce. Whilst the study documents a snapshot of only one region in England, the findings are salient to all dental practitioners and commissioners of NHS services who are seeking to progress new ways of working in general dental practice.

In keeping with other literature [27], we noted that there had been a considerable expansion of the dental workforce over the last 10 years, particularly in relation to the number of GDC-registered dental nurses and dental therapists working in the region. In the 2006 survey there was just one therapist per 39 dentists, whilst in 2016 there was one therapist per eight dentists, perhaps reflecting a greater interest in clinical DCP roles and associated increases in the number of undergraduate training places available through HEIs. However, whilst the figures indicate greater capacity for development of skill-mix within the region, this opportunity appears to be poorly realised. Similar findings reported in past surveys of these groups suggest little progress has been made over the decade [19, 27].

General Dental Practitioners attending the workshop acknowledged that the skills of dental therapists were sub-optimally deployed within NHS practices. In previous work [19], respondents attributed poor utilisation of dental therapists to dentists’ lack of awareness of their skills. By contrast, in this study workshop participants recognised the opportunities afforded by DCPs more widely delivering clinical care, but highlighted a number of practical barriers to their use.

These barriers included a lack of physical space in many practices. There was also a perception among some that patients may be reluctant to have treatment interventions performed by a DCP. While, in fact, there was little evidence of this being a problem that participants had actually experienced, the finding nonetheless indicates a need for dental practitioners to understand better public awareness of the roles and responsibilities of various dental professional groups, and their expectations of practice. Access to information and resources may afford patients greater choice in new models of care, whilst managing such expectations appropriately.

A prevailing concern related to feelings of uncertainty fuelled by the long-awaited reform of the 2006 NHS primary dental care contract in England. Ambiguity around the reformed contract reportedly stifled many (but not all) primary care dental practices from employing and embracing the full skill set of dental therapists as a distinct professional group. As new contract prototypes are piloted the implications for finances will become clear, but a unanimous view in this work was that skill-mix could only function with appropriate remuneration systems that recognise the ‘whole DCP package’, which has emphasis on patient education, disease prevention and oral health promotion.

A second key finding was a likely loss of expertise in the workforce through low retention of practitioners in older age groups. Survey data showed a continuing pattern of a sharp decline of workforce numbers (especially dental nurses) over 46 years of age. In keeping with past work [27], our participants suggested that this may reflect limited career progression in nursing, which causes work dissatisfaction.

Thirdly, there were high rates of part-time working amongst all registrant groups, but there had been a notable increase in part-time working among male dentists. One third of male dentists reported working part-time within a single practice in 2006, compared to around 80% in 2016. In this study, we did not determine whether this change was due to flexible working across roles, namely, individuals working full-time across different jobs, or if they were choosing to work fewer hours for an improved work-life balance. Other data generated by final year dental students and newly qualified dentists [28, 29] similarly indicated a preference for part-time working. The finding suggests that many dentists may now be opting for different patterns of working. It warrants further study to explore drivers to career choices, given the relationship with wellbeing [30] and the advantages of portfolio careers noted elsewhere in general medical practice [31].

Together these data highlight significant changes in the career intentions of dental professionals that require attention in future workforce planning.

### Limitations

The response rate of 53% for the questionnaire (228 of 431 primary care dental practices in the region) was impressive for a questionnaire of this length [32], and is in keeping with other recent postal workforce surveys in dentistry [33], but means that caution must be exercised in interpretation of the study’s findings.

Firstly, given the voluntary nature of study participation there was a risk of selection bias. We attempted to minimise this risk by clearly identifying our target population and means of recruitment, with guidance from the Project Advisory Group. All practices received prior notification of the study and were given opportunities to participate using either online, or paper questionnaires. A member of the study team also made direct contact with every non-responding practice in order to invite and support participation.

Response bias is a risk in any survey, although we received good response rates across the region, and across organisational sectors, suggesting no systematic bias. In addition, the numbers of individuals in each professional group represented in our data set were proportional to the total numbers of registrants in those groups on the GDC register in our region. This suggests that responding practices did not reflect any with a disproportionate representation of any professional group. Nonetheless, the composition of workforce in non-responding practices cannot be inferred.

There is also risk of reporting bias as the accuracy of self-reported responses from practices could not be validated against staff members’ actual working practises. However, as questionnaires were completed by staff who had relevant practice figures to hand, we have no reason to doubt the credibility of data.

It is possible that using the CQC database as a way of identifying sites meant we did not reach ‘non-clinical’ dental professionals (e.g. dental technicians) who may not always operate from a clinical dental practice address as they do not have direct patient contact. However, there are unlikely to be many in this group, and any omission will reflect a small variation around the small numbers we report.

Finally, the findings from this workforce survey and symposium are based upon a sample from one area in England, and hence the quantitative findings may not necessarily be transferable across other regions. For example, we have identified a higher proportion of respondents offering NHS services than in a survey of UK-wide practitioners conducted at a similar time [34]. Despite this potential limitation, the perspectives of our practitioners are likely to be applicable to other areas of the country where practices function under the same financial and NHS dental contractual regulations.

### National context

Since completion of this study, HEE has published its final report from a first phase of work entitled *‘Advancing Dental Care: Education and Training Review’* (the *ADC Review)* which has begun the process of identifying a future dental workforce model. This will be underpinned by review of the content and structure of education and training [14]. Phase 2 will include targeted engagement exercises involving dental students and other trainees. Hence, the factors influencing development of the dental team identified in this study are timely and salient to the national agenda by casting light on the realities of general dental practice experienced by our participants.

One of the conclusions drawn by the detailed national report from the Dental Workforce Advisory Group for England (DWAG) on ‘The future oral and dental workforce for England’ was the need to ‘*liberate the workforce*’ in order to meet future population needs [7]. However, the present study provides evidence that this point is some way off with dental professionals (particularly primary care dentists) highlighting numerous challenges in their attempts to optimise skill-mix.

### Future directions

This study has raised a number of important questions for workforce planning.

In particular, there are questions around the ‘life-course’ of a dental career: at the one end, questions arise around the experience of new dentists as they take on clinical complexity and adapt to a leadership role in the dental team. At the other, questions relate to retention of experienced dental staff who are being lost to the workforce in the mid-career stage. The morale of clinical dental professionals may be a revealing area for investigation and help support a ‘healthy’ and sustainable dental workforce. A need to improve morale has been highlighted by the recent 2019 dental workforce report for England, with recommendations for action, including a requirement for NHS contract reform alongside developing appropriate business models and payment systems [7].

Finally, to facilitate meaningful analysis in the future relating to skill-mix and the types of service being delivered by DCP groups to patients, we concur with the findings of others who have stressed that finer grain data is needed, particularly that the professional role of the individual clinician delivering each treatment or intervention should be coded and recorded [35].

## Conclusions

The composition and work patterns of the regional dental workforce in the North East of England and North Cumbria has changed markedly over the last 10 years. However, there are tensions and challenges experienced by NHS primary dental care providers who are attempting to enact and expand skill-mix. Fundamentally, despite its potential merit, skill-mix is not optimal within NHS primary dental care. Dental workforce planning needs to involve the whole dental profession, regulatory bodies, other health care sectors and educators if dental teams are to meet the changing needs of patients and populations in the future.

## Supplementary information

**Additional file 1.** Planning and developing the dental workforce of the future [26].

## Data Availability

The datasets used and analysed during the current study are available from the corresponding author on reasonable request.

## Abbreviations

ADC: Advancing Dental Care (HEE Review)
CQC: Care Quality Commission
DCP: Dental Care Professional
DWS: Dental Workforce Survey
EDDN: Extended-Duty Dental Nurse
EEA: European Economic Area
GDC: General Dental Council
GDP: General Dental Practitioner
GDS: General Dental Services
HEE: Health Education England
HEE-NENC: Health Education England working across the North east and North Cumbria
HEI: Higher Education Institution
NHS: National Health Service
PAG: Project Advisory Group
UDA: Unit of Dental Activity
