# Supplementary material for: Development and retention of the dental workforce: findings from a regional workforce survey and symposium in England
Source: BMC Health Serv Res. 2020 Mar 26;20:255. doi: 10.1186/s12913-020-4980-6 (PMC7099783; doi:10.1186/s12913-020-4980-6)
Supplement: Supplementary file 1 — Additional file 1. Planning and developing the dental workforce of the future [26]. [file 12913_2020_4980_MOESM1_ESM.docx]

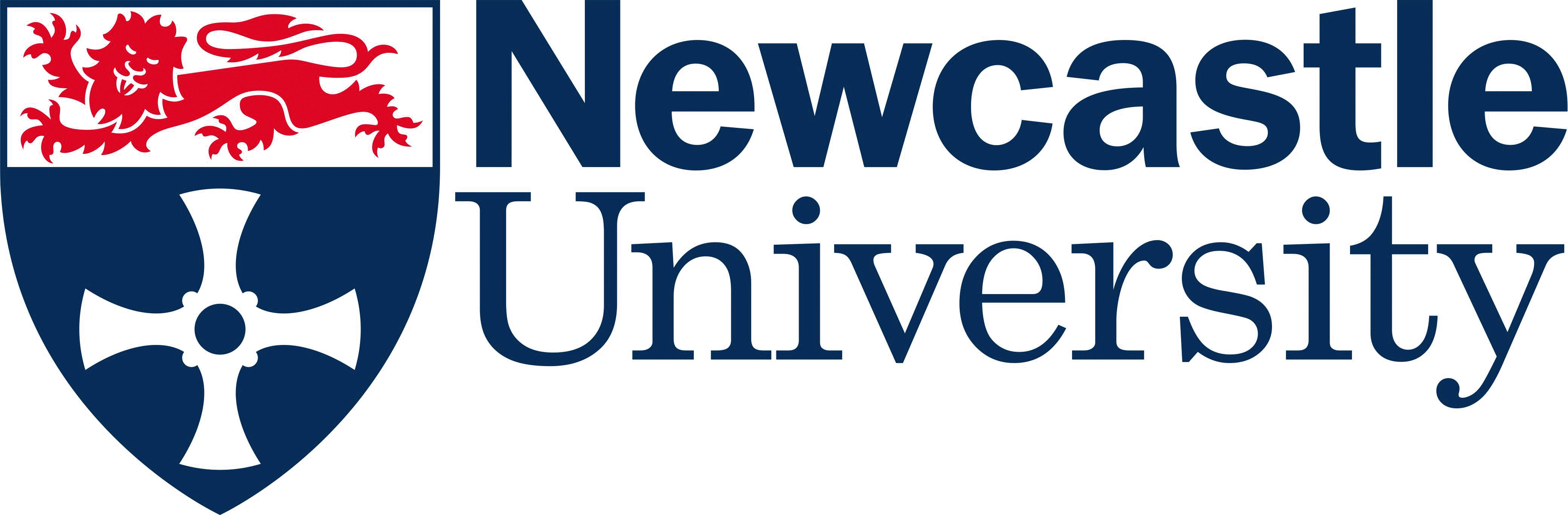
Dental Workforce Survey

**March 2016**

***Planning and developing the dental workforce of the future.***

| The Practice Manager  «Practice_Name»  «Address_1»  «Address_2»  «Address_3»  «Postcode» | If you would prefer to complete the survey online, please go to **www.tinyurl.com/DentalWorkforce2016** and enter this code when asked: «Q_ID»  If you have any queries, please contact **dentalworkforcesurvey@newcastle.ac.uk** |
| --- | --- |

Dear Colleague

Newcastle University Dental School and School of Medical Education are undertaking a study of the **private and NHS** **dental** **workforce across North East England and North Cumbria.**

This work will allow those responsible for commissioning the training of all dental healthcare professionals to plan for the workforce needs of the future, in **private as well as NHS sectors**.

The questionnaire should be completed with staff numbers for the whole practice based at the location named below (if part of a partnership or group, each location should return a questionnaire). Please correct any details of practice name or address before returning.

The enclosed FAQ provides more details of why some of this information is being collected.

Your participation in this work is very much appreciated.

Yours sincerely,


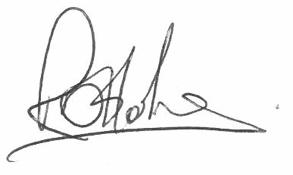

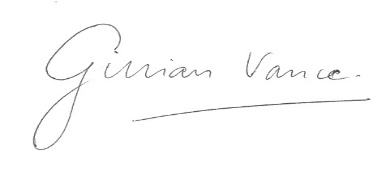


Richard Holmes Gillian Vance

Senior Lecturer/ Clinical Senior Lecturer

Hon Consultant in Dental Public Health *School of Medical Education*

*Newcastle Dental School*

| **Person completing survey**  Name: _______________________________  Position: _____________________________  Contact email: ________________________  Contact number: ______________________ | Please give these details so we can contact you in the unlikely circumstances that we require clarification. |
| --- | --- |

| 1. Does this practice treat patients:   ⃝ Exclusively privately  ⃝ Exclusively NHS  ⃝ A mixture of private and NHS;  please give approx. % NHS: _____ | 1. Is this practice independent or a member of a corporate/group provider?   ⃝ Independent (single owner)  ⃝ Independent (partnership)  ⃝ Corporate/group (please specify which)  ________________________ |
| --- | --- |

Staff numbers

**To plan the workforce requirements of the future, it is important to know the demographic composition of the current workforce – both private and NHS. For each of the groups below, please complete the requested information.**

**Dentists**

1. How many dentists work in your practice location? _____
2. For each dentist, excluding any visiting specialists, please give the following details.
   (If more than fifteen, please continue on the back of the questionnaire).

|  |  |  |  |  | **Which days are usually worked?** | | | | | | |  |
| --- | --- | --- | --- | --- | --- | --- | --- | --- | --- | --- | --- | --- |
|  | Sex | Age group:  1 – Under 30 2 – 30-45  3 – 46-55  4 – Over 55 | Normal weekly hours | Approx. % NHS workload | Mon | Tue | Wed | Thu | Fri | Sat | Sun | Is this a Foundation dentist  (please tick) |
| **Dentist 1** |  |  |  |  |  |  |  |  |  |  |  |  |
| **Dentist 2** |  |  |  |  |  |  |  |  |  |  |  |  |
| **Dentist 3** |  |  |  |  |  |  |  |  |  |  |  |  |
| **Dentist 4** |  |  |  |  |  |  |  |  |  |  |  |  |
| **Dentist 5** |  |  |  |  |  |  |  |  |  |  |  |  |
| **Dentist 6** |  |  |  |  |  |  |  |  |  |  |  |  |
| **Dentist 7** |  |  |  |  |  |  |  |  |  |  |  |  |
| **Dentist 8** |  |  |  |  |  |  |  |  |  |  |  |  |
| **Dentist 9** |  |  |  |  |  |  |  |  |  |  |  |  |
| **Dentist 10** |  |  |  |  |  |  |  |  |  |  |  |  |
| **Dentist 11** |  |  |  |  |  |  |  |  |  |  |  |  |
| **Dentist 12** |  |  |  |  |  |  |  |  |  |  |  |  |
| **Dentist 13** |  |  |  |  |  |  |  |  |  |  |  |  |
| **Dentist 14** |  |  |  |  |  |  |  |  |  |  |  |  |
| **Dentist 15** |  |  |  |  |  |  |  |  |  |  |  |  |

1. Please list any additional postgraduate qualifications held by dentists working in this practice (eg MSc, MFDS, MJDF)

|  |
| --- |

| 1. How many visiting specialists attend this practice location? |  |
| --- | --- |

1. Which services do they provide and how often?

|  |  |
| --- | --- |

**Other staff**

1. In the following tables, please complete the requested information for each staff group.
   If there are no staff in a given group, please write ‘None’ in the top row.

|  | **a) Dental therapists** | | **b) Dental hygienists** | | **c) GDC registered dental nurses*** | | **d) Dental nurses in training** | |
| --- | --- | --- | --- | --- | --- | --- | --- | --- |
|  | Male | Female | Male | Female | Male | Female | Male | Female |
| **Number of staff** |  |  |  |  |  |  |  |  |
| **Total hours worked in this location (approx.)** |  |  |  |  |  |  |  |  |
| **Number aged under 30** |  |  |  |  |  |  |  |  |
| **Number aged 30-45** |  |  |  |  |  |  |  |  |
| **Number aged 46-55** |  |  |  |  |  |  |  |  |
| **Number aged over 55** |  |  |  |  |  |  |  |  |

*** Excluding qualified dental nurses whose main role is in another job – eg practice manager or receptionist.**

|  | **e) Clinical dental technicians** | | **f) Dental technicians** | | **g) Orthodontic therapists** | | **h) Practice managers** | | **i) Receptionists** | |
| --- | --- | --- | --- | --- | --- | --- | --- | --- | --- | --- |
|  | Male | Female | Male | Female | Male | Female | Male | Female | Male | Female |
| **Number of staff** |  |  |  |  |  |  |  |  |  |  |
| **Total hours worked in this location (approx.)** |  |  |  |  |  |  |  |  |  |  |
| **Number aged under 30** |  |  |  |  |  |  |  |  |  |  |
| **Number aged 30-45** |  |  |  |  |  |  |  |  |  |  |
| **Number aged 46-55** |  |  |  |  |  |  |  |  |  |  |
| **Number aged over 55** |  |  |  |  |  |  |  |  |  |  |

| 1. How many of the dental nurses in this practice counted in c) above also regularly have other roles in the practice (eg reception or practice manager – not counting ad hoc sickness cover etc.)? | |  |
| --- | --- | --- |
| 1. Is the practice manager a qualified dental professional? | ⃝ Yes  ⃝ No | |

1. Please list any current vacancies in any of the above staff groups.

|  |
| --- |

Place of primary dental qualification

1. We would like to know how much of the local workforce qualified locally and elsewhere.
   Please enter the numbers of staff in each group who obtained their primary dental qualification in each area.

|  | **Qualified in North East England** | **Qualified elsewhere in the UK** | **Qualified elsewhere in the European Economic Area*** | **Qualified elsewhere in the world** | **Not known** |
| --- | --- | --- | --- | --- | --- |
| **Dentists** |  |  |  |  |  |
| **Dental therapists** |  |  |  |  |  |
| **Dental hygienists** |  |  |  |  |  |
| **Dental nurses** |  |  |  |  |  |
| **Clinical dental technicians** |  |  |  |  |  |
| **Dental technicians** |  |  |  |  |  |
| **Orthodontic therapists** |  |  |  |  |  |

* Austria, Belgium, Bulgaria, Croatia, Cyprus, Czech Republic, Denmark, Estonia, Finland, France, Germany, Greece, Hungary, Iceland, Ireland, Italy, Latvia, Liechtenstein, Lithuania, Luxembourg, Malta, Norway, Netherlands, Poland, Portugal, Romania, Slovakia, Slovenia, Spain, Sweden or Switzerland.

Dental nurse qualifications

**For these questions, ‘dental nurses’ includes all qualified nurses in the practice, even if their primary role is in another job.**

1. How many dental nurses in this practice location have or are in training for the following primary dental qualifications?

|  | Have qualification | In training |
| --- | --- | --- |
| **National Examining Board for Dental Nurses (NEBDN) National Diploma in Dental Nursing** |  |  |
| **City and Guilds NVQ Level 3 diploma in Dental Nursing** |  |  |
| **Certificate of Higher Education in Dental Nursing** |  |  |

| 1. How many dental nurses working at this practice location are qualified as Extended Duties Dental Nurses (EDDNs)? |  |
| --- | --- |

1. How many dental nurses in this practice location have or are in training for the following specialised NEBDN qualifications?

|  | Have qualification | In training |
| --- | --- | --- |
| **Dental Implant Nursing** |  |  |
| **Dental Radiography** |  |  |
| **Dental Sedation Nursing** |  |  |
| **Oral Health Education** |  |  |
| **Orthodontic Dental Nursing** |  |  |
| **Special Care Dental Nursing** |  |  |

Activities performed by different staff groups

1. Please tick which of the following activities are undertaken by the following staff groups at any time in this practice location? Please consult with the Principal Dentist or Practice owner to complete this table if necessary.

|  | Dental Therapist | Dental Hygienist | Dental Nurse (including joint receptionist or practice manager roles) | Clinical Dental Technician | Dental Technician | Orthodontic Therapist |
| --- | --- | --- | --- | --- | --- | --- |
| **Prescribe radiographs** |  |  |  |  |  |  |
| **Take radiographs** |  |  |  |  |  |  |
| **Apply fluoride varnish to teeth** |  |  |  |  |  |  |
| **Deliver Oral Health Education** |  |  |  |  |  |  |
| **Take impressions** |  |  |  |  |  |  |
| **Give smoking cessation advice** |  |  |  |  |  |  |
| **Measure and record plaque indices** |  |  |  |  |  |  |
| **Administer inhalation sedation** |  |  |  |  |  |  |
| **Rubber dam** |  |  |  |  |  |  |
| **Cannulation** |  |  |  |  |  |  |

| 1. Approximately what proportion (as a percentage) of dental therapists’ time is spent performing hygienist activities or competencies? |  |
| --- | --- |

1. Which of the following specialised services are offered by dentists in this practice location (please tick)?

| **Implants** |  | **IV sedation** | | |  | |
| --- | --- | --- | --- | --- | --- | --- |
| **Orthodontic services** |  | Inhalation sedation | | |  | |
| **Minor oral surgery (excluding simple exodontia)** |  | Domiciliary care | | |  | |
| **Other (please specify)** | | |  |  | |  |

1. Please list any development or learning needs that you have identified among your staff. For example, if relevant, what might encourage you to become a foundation training practice? Please identify as much detail as you think is relevant.

|  |
| --- |

1. Finally, please add any other information that may help us interpret the shape and needs of the dental workforce. For example, what growth or future employment plans may you have in the practice?

|  |
| --- |

Thank you for completing the questionnaire.

Question 4 continuation table

|  |  |  |  |  | **Which days are usually worked?** | | | | | | |  |
| --- | --- | --- | --- | --- | --- | --- | --- | --- | --- | --- | --- | --- |
|  | Sex | Age group:  1 - Under 30 2 - 30-55  3 - Over 55 | Weekly hours | Approx. % NHS workload | Mon | Tue | Wed | Thu | Fri | Sat | Sun | Is this a Foundation dentist  (please tick) |
| **Dentist 16** |  |  |  |  |  |  |  |  |  |  |  |  |
| **Dentist 17** |  |  |  |  |  |  |  |  |  |  |  |  |
| **Dentist 18** |  |  |  |  |  |  |  |  |  |  |  |  |
| **Dentist 19** |  |  |  |  |  |  |  |  |  |  |  |  |
| **Dentist 20** |  |  |  |  |  |  |  |  |  |  |  |  |
| **Dentist 21** |  |  |  |  |  |  |  |  |  |  |  |  |
| **Dentist 22** |  |  |  |  |  |  |  |  |  |  |  |  |
| **Dentist 23** |  |  |  |  |  |  |  |  |  |  |  |  |
| **Dentist 24** |  |  |  |  |  |  |  |  |  |  |  |  |
| **Dentist 25** |  |  |  |  |  |  |  |  |  |  |  |  |

*The Dental Workforce Survey 2016 is conducted by Newcastle University. All data will be held securely at Newcastle University.
A report containing aggregated anonymous data will be provided to Health Education England.*
